# Potential effects of beta-blockers in HFpEF

**DOI:** 10.1007/s10741-024-10468-w

**Published:** 2024-12-03

**Authors:** Wojciech Tokarczyk, Szymon Urban, Patryk Patrzałek, Łukasz Stolarski, Gracjan Iwanek, Oskar Szymański, Robert Zymliński

**Affiliations:** 1 Institute of Heart Diseases, University Hospital in Wrocław, Wrocław, Poland; 2 Institute of Heart Diseases, University Hospital in Wrocław, Wrocław Medical University, Wrocław, Poland; 3 District Hospital in Rawicz, University Hospital in Wrocław, Wrocław Medical University, Wrocław, Poland; 4 District Hospital in Rawicz, Rawicz, Poland

**Keywords:** Heart failure, HFpEF, Beta-blockers, Pharmacotherapy, Sympathetic activity

## Abstract

Heart failure with preserved ejection fraction (HFpEF) poses a significant challenge in contemporary medicine, characterized by poor quality of life, high healthcare costs, and increased mortality. Despite advancements in medical research, treatment strategies for HFpEF remain elusive, with unclear guidance on the use of beta-blockers. While sympathetic overstimulation is common in HFpEF, beta-blockers, though potentially beneficial in reducing sympathetic activity, may exacerbate chronotropic incompetence and decrease exercise tolerance. Additionally, their impact on outcomes in HFpEF patients with concurrent atrial fibrillation is uncertain. Some studies suggest the potential benefits of beta-blockers on diastolic function, yet evidence on clinical endpoints remains inconclusive. Recent research indicates a potential reduction in all-cause mortality with beta-blocker use in HFpEF, although their effect on combined mortality or HF hospitalizations is less clear. Moreover, beta-blocker efficacy may vary depending on ejection fraction subgroups, with more favorable outcomes observed in HFmrEF compared to HFpEF. Current literature underscores the need for large-scale randomized clinical trials to clarify the role of beta-blockers in HFpEF management. Given the limitations of existing evidence, future research is essential to inform updated treatment guidelines and therapeutic protocols tailored to the contemporary clinical landscape.

## Introduction/guidelines

Heart failure (HF) continues to stand as a paramount challenge within contemporary medicine. Despite considerable strides in medical research, some questions are left without any answer. HFpEF is a clinical syndrome associated with poor quality of life, high healthcare expenses, and premature mortality [1]. The variability of HFpEF contributes to the challenges encountered in implementing effective management strategies on a wide scale. Guidelines [2] define patients with HF with preserved ejection fraction as those with symptoms and signs of HF, with evidence of structural and/or functional cardiac abnormalities and/or raised natriuretic peptides, and with a left ventricle ejection fraction (LVEF) ≥ 50%.

The 2021 ESC guidelines [2] indicate that 80% of HFpEF patients are prescribed beta-blockers for indications other than HFpEF, i.e., ischemic heart disease, hypertension, arrhythmias, hyperthyroidism, and so on. Beta-blockers are not considered in the 2021 ESC guidelines for HFpEF treatment, except when patients are already taking them for concomitant indications that coexist with HFpEF. In the 2023 update on HFpEF treatment, only dapa-/empagliflozin, diuretics in case of retention, and the management of comorbidities are mentioned with a Class I recommendation. This leaves the primary document guiding treatment without a clear conclusion regarding the appropriateness of beta-blocker use. Given this, we decided to perform a comprehensive literature review to present the current evidence.

## Sympathetic activity

As a physiological compensating mechanism during the course of HF, the sympathetic nervous system becomes hyperactive. The consequent elevation in sympathetic activity initially results in a positive inotropic effect. Yet, sustained beta-receptor stimulation causes immoderate cardiomyocyte apoptosis and promotes hypertrophy, fibrosis, and dysfunctional remodeling of cardiac muscle tissue. Increased sympathetic activity in HFpEF might be a promising therapeutic target point supporting beta-blocker use in this indication [3].

The adaptability of cardiac function to immediate demands predominantly hinges on the physiological regulation of the autonomic nervous system. Instances characterized by inadequate elevation of heart rate lead to the manifestation of chronotropic incompetence, which clinically presents as exercise intolerance. This condition markedly diminishes patients’ quality of life and stands as an autonomous prognostic factor for significant adverse cardiovascular events and overall mortality [4].

Regrettably, research indicates that the use of beta-blockers influences the exacerbation of chronotropic incompetence to a significant extent [5]. Moreover, beta-blocker withdrawal might be beneficial to exercise capacity in patients with HFpEF with a decreased indexed LV end-systolic volume as a potential marker for individuals who are more likely to exhibit significant short-term improvements in maximal functional capacity following the discontinuation of beta-blocker therapy [6]. Goyal et al. [7] in their article discuss the innovative application of N-of-1 trials in the context of deprescribing beta-blockers for patients with HFpEF. The potential of these trials is to address uncertainties about the risks and benefits of deprescribing, offering a personalized approach to medication management that considers both physiological and psychological factors, ultimately aiming to improve patient outcomes through data-driven decision-making [7]. Noteworthy, a recent study sought to determine whether restoring exertional heart rate through atrial pacing would be beneficial for patients with chronotropic incompetence in HFpEF had negative results, i.e., atrial pacing demonstrated no significant effect on cardiac output during exercise, attributable to a concomitant decrease in stroke volume [8].

One can infer that discontinuation of beta-blocker therapy should be favored over pacemaker implantation in these patients.

## Atrial fibrillation

HFpEF frequently coexists with atrial fibrillation. Notably, contemporary guidelines advocate beta-blockers or non-dihydropyridine calcium channel blockers as the primary pharmacotherapeutic agents for ventricle rhythm rate control in atrial fibrillation. Administration of beta-blockers in this context manifested in an elevation of NT-pro BNP concentrations, both at rest and during peak exercise, relative to baseline values. In contrast, treatment with diltiazem and verapamil markedly lowered NT-pro BNP levels [9]. The controversy surrounding the upsurge in natriuretic peptide concentrations as a parameter for monitoring heart failure progression persists. The augmentation in natriuretic peptide levels during beta-blocker therapy is believed to emanate from their pharmacological influence, notably in the context of reducing heart rate, prolonging cardiac filling times, and negative inotropic effects, consequently, this affects left ventricular wall stress and exerts an effect on NT-pro BNP secretion [10].

There is a lack of consistent evidence supporting prognostic benefits in beta-blocker administration for individuals with both heart failure and concurrent atrial fibrillation [11].

## Diastolic function

Left ventricle (LV) diastolic dysfunction defined as an impairment in relaxation, increased chamber wall stiffness, or a combination of the two, leads to symptomatic HF by causing abnormally high filling pressures at rest and/or during exercise [12].

Anomalous mechanical characteristics of the myocardium imply elevated ventricular filling pressures; consequently, the dependency on atrial contraction increases to maintain filling and cardiac output. In the context of previously discussed concurrent atrial fibrillation where the late-filling wave of ventricles is negligible, the result is hemodynamically highly unfavorable.

Elevated filling pressures exacerbate symptoms of dyspnea reduce exercise capacity [13, 14] and, most importantly, elevate the risk of HF hospitalization [15] and decrease survival in HFpEF [16].

That engenders optimism regarding the potential impact of beta-blockers on the hemodynamics of patients with HFpEF and the associated prospective advantages.

Heart rate reduction due to beta-blockade ameliorates the left ventricle filling by increasing the diastolic time, which also improves myocardial perfusion and metabolism.

The SWEDIC study, a randomized, double-blinded multi-center study focused on diastolic function and included patients with EF > 40%, due to old terminology they were considered as a group with diastolic heart failure (DHF). The study sought to determine whether non-selective beta-receptor blockade and selective vasodilating alpha-1 receptor blockade of carvedilol could play a significant role in the DHF treatment. The results showed that based on the E/A ratio which was the most useful indicator of diastolic function (DF) improvement, the treatment with carvedilol turned out to improve DF, and the effect was more pronounced in patients with initial higher heart rates. However, despite improvement in diastolic function, no statistically significant change in NYHA class or reduction in hospitalizations was observed [17].

## The effect on outcome

The potential effects discussed in the previous paragraphs may often be difficult to distinguish clinically and therefore be less convincing. Clinical endpoints are of paramount importance in assessing measurable impact significance and the impact resulting from the integration of therapeutic modalities into the healthcare system. Therefore, we have decided to emphasize the research focusing on the endpoints disregarding the minor effects. Translating into the currently prevailing terminology based on 2021 ESC guidelines [2], patients undergoing the aforementioned analysis of the SWEDIC study [17] can be divided into the HFmrEF (EF 41–49%) and HFpEF (EF ≥ 50%) subgroups. The results of a recent study showed that treatment with beta-blockers may be associated with reduced risk of hospitalization and death only in the HFmrEF patient group. In the HFpEF group, there is a lack of significant reduction in the risk of hospitalization, or survival benefit, particularly in the subgroup with an EF > 60% [18].

The study focusing on the association between the use of beta-blockers and different outcomes in patients with HFpEF revealed that the use of beta-blockers was associated with lower all-cause mortality but did not reduce combined all-cause mortality or HF hospitalizations. With a median follow-up of 709 days in the matched cohort, 1-year survival was 80% vs 79% for treated vs untreated patients, and 5-year survival was 45% vs 42%, with 2279 (41%) vs 1244 (45%) total deaths and 177 vs 191 deaths per 1000 patient-years; moreover, beta-blockers use did not reduce combined mortality or heart failure hospitalizations—3368 (61%) vs 1753 (64%) total for first events, with 371 vs 378 first events per 1000 patient-years. To highlight the differences between the different phenotypes of HF, it is worth mentioning that in the matched HFrEF cohort, beta-blockers reduced mortality and also reduced combined mortality or heart failure hospitalization [19].

In the fully adjusted first model of the TOPCAT trial analysis, patients with an ejection fraction of 50% or greater who were receiving beta-blockers had a higher relative risk of heart failure hospitalization compared to those not receiving beta-blockers. In a second sensitivity analysis involving propensity score-matched cohorts, beta-blocker use was also associated with a higher number of hospitalizations due to heart failure among patients with an ejection fraction of 50% or greater [20].

The SENIORS trial with nebivolol [21] included HF patients regardless of EF and demonstrated a beneficial beta-blocker effect independent of EF [22]. In a meta-analysis comprising 25 RCTs, beta-blockers exhibited a decrease in both all-cause and cardiovascular mortality when compared to a placebo in patients with an ejection fraction greater than 40% (The American College of Cardiology defined a group of patients with 41 to 49% EF as borderline HFpEF at the time of this meta-analysis being undertaken) [23]. The value of this study in assessing the use of beta-blockers for treating HFpEF may be limited due to the inclusion criteria; in the 25 studies analyzed, 7 studies included patients with EF > 40%, 9 studies included patients with EF > 45%, and only 9 studies focused on patients with EF > 50%. Additionally, some of the studies had only a 12-week follow-up period.

The current scientific discourse emphasizes the importance of both duration and dose in the context of beta-blocker administration [24].

The findings from the J-DHF study indicate that the administration of the standard carvedilol dose of 7.5 mg/day in patients with EJ ≥ 50% did not demonstrate a reduction of the risk of any composite outcomes. However, the risks for a composite of cardiovascular or all-cause death and unplanned hospitalization for any cardiovascular causes tended to be reduced when compared with the control group [25].

An analysis of the Medicare-linked OPTIMIZE-HF data, that included 26,376 patients, of which 8873 had HFpEF, defined as ejection fraction ≥ 50%, revealed that among hospitalized elderly patients with HFpEF and a discharge heart rate > 70 bpm, the utilization of high-dose beta-blockers is linked to a reduced risk of all-cause readmission and the combined endpoint of all-cause readmission or all-cause mortality; nevertheless, the use of high-dose beta-blockers did not translate to a significant reduction of HF hospital readmissions [26].

In the context of the evaluation of a nationwide sample of 13,533 applicable patients with the diagnosis of HFpEF, the administration of beta-blocker therapy revealed a non-significant trend in survival benefits after 1 year. However, a statistically significant improvement in survival outcomes was observed after 3 years of treatment. The observed reduction in mortality with beta-blockers, when contrasted with the more pronounced effects of statins and renin–angiotensin–aldosterone system inhibitors, suggests a substantial proportion of patients with underlying coronary artery disease. This highlights the heterogeneity of HFpEF populations in older cohorts and raises important considerations about the potential confounding effects of comorbidities such as coronary artery disease on these outcomes [27].

Yndigegn et al. [28] in their recently published study highlighted that the widespread use of beta-blockers post-myocardial infarction is based on research conducted prior to the era of biomarker-driven diagnostics and intravascular interventions and modern pharmaceuticals, questioning their current relevance. A randomized clinical trial aimed at assessing the impact of long-term beta-blocker therapy in patients post-myocardial infarction with accompanying HFpEF revealed that patients treated according to current guidelines did not derive benefit compared to the group not receiving beta-blockers, considering the composite primary end point of death from any cause or new myocardial infarction [28].

In a single-center retrospective cohort study of 20,206 patients with left ventricular ejection fraction (EF) ≥ 50% hospitalized for decompensated heart failure, beta-blocker therapy was associated with reduced all-cause mortality at 30 days, 1 year, and 3 years (*p* < 0.0001). This mortality benefit was further confirmed through a propensity score-matched analysis. Additionally, a secondary analysis demonstrated that the combination of beta-blockers with either spironolactone (*p* = 0.0359) or ACEi/ARBs (*p* < 0.0001) significantly reduced mortality at 3 years, even in patients with comorbid conditions such as hypertension, coronary artery disease, or atrial fibrillation [29].

The STRONG-HF trial was terminated early due to its remarkable results, demonstrating significant benefits of intensive treatment. It included patients across the spectrum of LVEF, including those with HFpEF. Beta-blockers were up-titrated more aggressively in the high-intensity care group, with 49% of patients reaching full doses by day 90 compared to 4% in the usual care group. Subgroup analysis showed that the intensive treatment strategy, including beta-s, was equally effective in patients with HFpEF as in those with HFrEF or HFmrEF. The study also demonstrated a significant reduction in the primary endpoint (180-day heart failure readmission or all-cause death), suggesting the potential benefits of beta-blockers in HFpEF when up-titrated effectively [30].

It is important to keep in mind that RCTs have shown that mortality decreases as ejection fraction improves. This contrasts with heart failure with HFpEF, where mortality is largely driven by non-cardiovascular causes, emphasizing the complex and multifactorial nature of the condition. Consequently, most studies prioritize total mortality rather than focusing solely on cardiovascular mortality.

To provide a clearer representation of the cited study results, they have been consolidated into Table 1.

**Table 1:** Summary of overall effects of beta-blockers use in HFpEF reported in analyzed studies

*Study*	*Type of study*	*Year*	*n*	*Setting*	*Substance*	*Effect on mortality*	*Other effects*
*Silverman *et al	RCT	2019	1761 (LVEF ≥ 50% *n* = 1567)	Clinical DHF; MI, 20%; AF, 42%; hypertension, 90%; LVEF ≥ 45%	NR	CV mortality (HR, 1.24; [95% CI, 0.85–1.79])	Higher risk for incident HF hospitalization (HR, 1.74 [95% CI, 1.28–2.37])
*Yndigegn *et al	RCT	2024	5020	MI; LVEF > 50%	Metoprolol, bisoprolol	NR	Neutral effect on the composite primary end point of death from any cause or new myocardial infarction (HR, 0.96; [95% CI, 0.79–1.16])
*Yamamoto *et al	RCT	2013	225 (LVEF ≥ 50% *n* = 209)	NYHA I–IV; AF, 37%; hypertension, 88%; ischaemic heart disease, 28%; LVEF > 40%	Carvedilol	NR	Neutral effect on the primary composite outcome, i.e., composite of cardiovascular death and unplanned hospitalization for heart failure (HR, 0.902; [95% CI, 0.546–1.488])
*Mebazaa *et al	RCT	2022	1075 (LVEF ≥ 50% *n* = 163)	AHF	NR	NR	Reduction of 180-day heart failure readmission or all-cause mortality (RR, 0.66 [95% CI, 0.50–0.86])
*Arnold *et al	Obs	2023	360,223	EF ≥ 40%	NR	Mortality; LVEF 50–59% (HR, 0.92; [95% CI, 0.89–0.95]); LVEF 60–69% (HR, 0.97; [95% CI, 0.94–0.99]); LVEF ≥ 70% (HR, 0.98; [95% CI, 0.93–1.03])	Higher risk of HF hospitalization; LVEF 50–59% (HR, 0.92; [95% CI, 0.88–0.97]); LVEF 60–69% (HR, 1.10; [95% CI, 1.05–1.16]); LVEF ≥ 70% (HR, 1.19; [95% CI, 1.08–1.30])
*Lund *et al	Obs	2014	8244 (LVEF ≥ 50% *n* = 4813)	NYHA I–IV; MI, 30%; AF, 50%; LVEF ≥ 40%	NR	Risk of all-cause mortality (HR, 0.93; [95% CI, 0.86–0.996])	No reduction in combined all-cause mortality or heart failure hospitalization (HR, 0.98; [95% CI, 0.92–1.04])
*Lam *et al	Obs	2018	8873	LVEF > 50%	Carvedilol, metoprolol, bisoprolol, atenolol	Risk of all-cause mortality (HR, 0.86; [95% CI, 0.75–0.98])	Positive effect on the combined endpoint of all-cause readmission or all-cause mortality (HR, 0.89; [95% CI, 0.80–1.00])
*Shah *et al	Obs	2008	13,533	Age ≥ 65; LVEF > 50%	NR	NR	Improvement in survival outcomes after 3 years of treatment (RR, 0.92; [95% CI, 0.87–0.97])
*Ibrahim *et al	Obs	2024	20,206	LVEF ≥ 50%; DHF;	Carvedilol; metoprolol	All-cause mortality (HR, 0.77; [95% CI, 0.66–0.90])	NR
*Cleland *et al	Meta	2018	314	HF; AF;	NR	All-cause mortality: AF (*n* = 73), (HR, 0.86; [95% CI, 0.19–3.94]) Sinus rhythm (*n* = 241) (HR, 1.79; [95% CI, 0.78–4.10])	Effect on LVEF; AF (*n* = 59) mean difference (SE); − 2.2% (3%) Sinus rhythm (*n* = 201) mean difference (SE); + 0.1% (1.2%)
*Zheng *et al	Meta	2018	1299	LVEF ≥ 40%	NR	All-cause mortality (RR, 0.78; [95% CI, 0.65–0.94]) and CV mortality (RR, 0.75; [95% CI, 0.60–0.94])	HF hospitalizations (HR 0.67; (95% CI, 0.42–1.07])

*n* number of patients, *NR* not reported, *EF* ejection fraction, *LVEF* left ventricle ejection fraction, *HF* heart failure, *HR* hazard ratio, *RR* risk ratio, *SE* standard error of the mean difference, *MI* myocardial infarction, *AF* atrial fibrillation, *DHF* decompensated heart failure, *CV* cardiovascular, *RCT* randomized controlled trial, *Obs* observational, *Meta* meta-analysis

It is noteworthy that certain referenced studies originate from periods during which alternative classifications and guidelines were in force, thus constraining their contemporary applicability [31–36].

## Conclusions

Given the current state of knowledge, it is challenging to draw specific conclusions that form the basis for changes in recommendations and therapeutic protocols. Furthermore, the role of beta-blockers in the context of HFpEF therapy, while holding promising prospects due to their multifaceted actions, does not yield clear conclusions regarding the endpoints considered in clinical trials. Current randomized clinical trials involving large patient cohorts are necessary to derive unequivocal conclusions about the efficacy of beta-blockers. Additionally, the evidence upon which we rely may already be outdated and was situated in a different clinical setting; therefore, new evidence is necessary. Future studies should focus on identifying specific HFpEF phenotypes that might respond better to beta-blockers. Stratifying trials based on comorbidities and clinical characteristics will be key in understanding the population that stands to gain the most. Future trials should focus on both hard endpoints (e.g., mortality, hospitalization rates) as well as soft endpoints (e.g., quality of life, exercise capacity). Given the heterogeneity of HFpEF patients, N-of-1 trials, where individual patients serve as their own control, could be valuable. These trials can help tailor beta-blocker therapy based on individual responses, particularly in a population where one-size-fits-all approaches may not be optimal. It is worth mentioning that future studies should include detailed safety monitoring protocols to assess the risks of withdrawal, including the potential for rebound effects such as tachycardia, hypertension, and worsening heart failure symptoms. The development of structured withdrawal protocols could help mitigate these risks and identify patients for whom continued beta-blocker therapy is necessary. A significant number of physicians initiate beta-blockers in HFpEF patients, often without evidence-based indications, and are frequently unwilling to deprescribe due to concerns about interfering with other physicians’ treatment plans. Given the potential for beta-blockers to cause harm in HFpEF, future research should also focus on developing approaches to improve prescribing practices and enhance physician-to-physician communication [37].

**Fig. 1:**
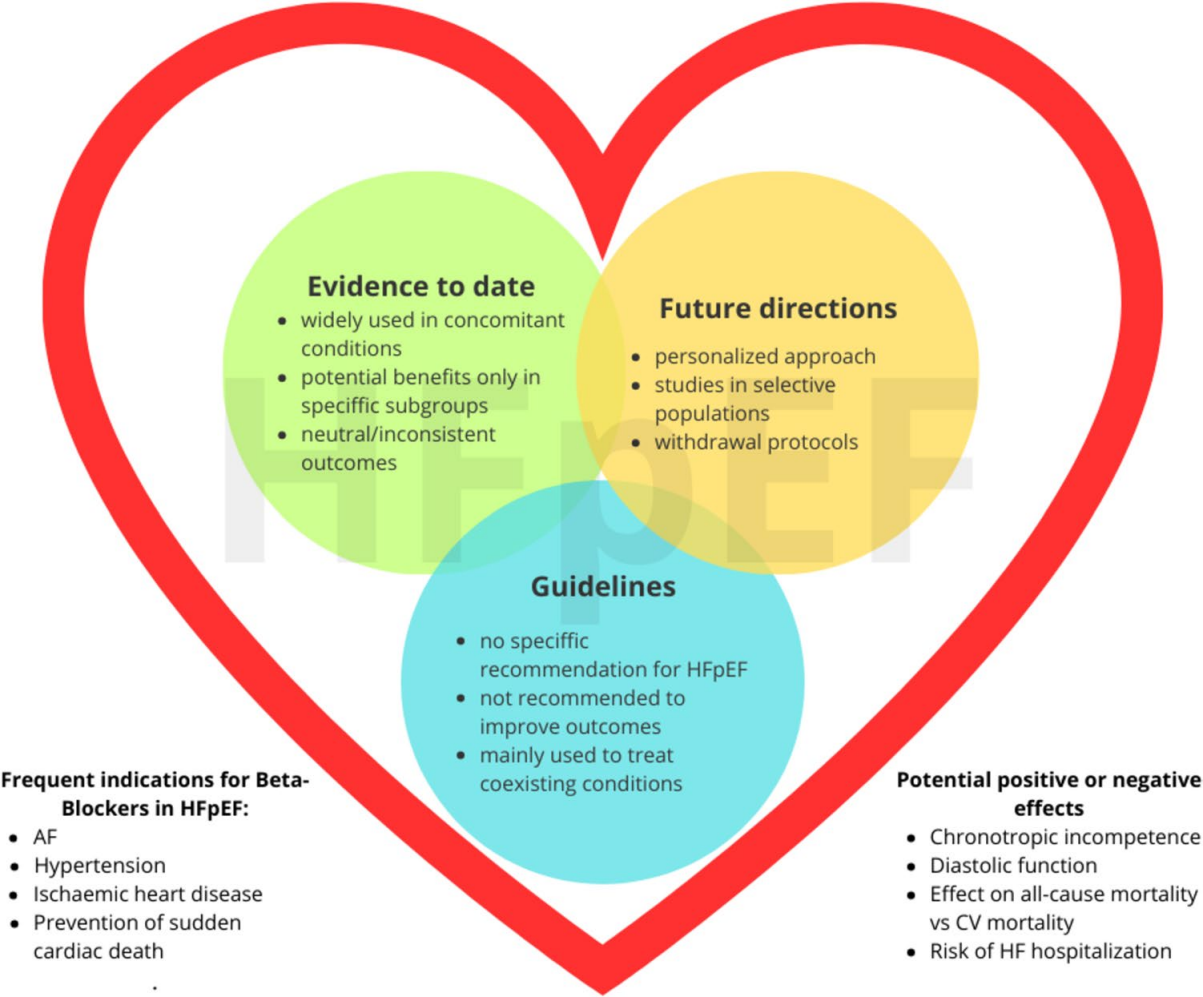
Illustration bridging together the guidelines, evidence to date, and future directions

Figure 1 is a graphical diagram illustrating the most pressing topics and conclusions from the three pillars of our discussion: guidelines, literature data, and future perspectives.

## References

[1] Dunlay SM, Roger VL, Redfield MM (2017) Epidemiology of heart failure with preserved ejection fraction. Nat Rev Cardiol 14(10):591–602. 10.1038/NRCARDIO.2017.65 28492288 10.1038/nrcardio.2017.65

[2] McDonagh TA et al (2021) 2021 ESC Guidelines for the diagnosis and treatment of acute and chronic heart failure. Eur Heart J 42(36):3599–3726. 10.1093/EURHEARTJ/EHAB368 34447992 10.1093/eurheartj/ehab368

[3] Grassi G, Mancia G, Esler M (2022) Central and peripheral sympathetic activation in heart failure. Cardiovasc Res 118(8):1857–1871. 10.1093/CVR/CVAB222 34240147 10.1093/cvr/cvab222

[4] Zweerink A, van der Lingen ALCJ, Handoko ML, van Rossum AC, Allaart CP (2018) Chronotropic incompetence in chronic heart failure. Circ Heart Fail 11(8):e004969. 10.1161/CIRCHEARTFAILURE.118.004969 30354566 10.1161/CIRCHEARTFAILURE.118.004969

[5] Brubaker PH, Kitzman DW (2011) Chronotropic incompetence: causes, consequences, and management. Circulation 123(9):1010–1020. 10.1161/CIRCULATIONAHA.110.940577 21382903 10.1161/CIRCULATIONAHA.110.940577 PMC3065291

[6] Palau P et al (2024) β-blocker withdrawal and functional capacity improvement in patients with heart failure with preserved ejection fraction. JAMA Cardiol 9(4):392. 10.1001/jamacardio.2023.5500 38324280 10.1001/jamacardio.2023.5500 PMC10851133

[7] Goyal P et al (2022) N-of-1 trials to facilitate evidence-based deprescribing: rationale and case study. Br J Clin Pharmacol 88(10):4460–4473. 10.1111/bcp.15442 35705532 10.1111/bcp.15442 PMC9464693

[8] Reddy YNV et al (2023) Rate-adaptive atrial pacing for heart failure with preserved ejection fraction: the RAPID-HF randomized clinical trial. JAMA 329(10):801–809. 10.1001/JAMA.2023.0675 36871285 10.1001/jama.2023.0675 PMC9986839

[9] Ulimoen SR et al (2014) Calcium channel blockers improve exercise capacity and reduce N-terminal Pro-B-type natriuretic peptide levels compared with beta-blockers in patients with permanent atrial fibrillation. Eur Heart J 35(8):517–523. 10.1093/EURHEARTJ/EHT429 24135831 10.1093/eurheartj/eht429

[10] Olsen MH et al (2006) N-terminal brain natriuretic peptide predicted cardiovascular events stronger than high-sensitivity C-reactive protein in hypertension: a LIFE substudy. J Hypertens 24(8):1531–1539. 10.1097/01.HJH.0000239288.10013.04 16877955 10.1097/01.hjh.0000239288.10013.04

[11] Cleland JGF et al (2018) Beta-blockers for heart failure with reduced, mid-range, and preserved ejection fraction: an individual patient-level analysis of double-blind randomized trials. Eur Heart J 39(1):26–35. 10.1093/EURHEARTJ/EHX564 29040525 10.1093/eurheartj/ehx564 PMC5837435

[12] Borlaug BA (2014) The pathophysiology of heart failure with preserved ejection fraction. Nat Rev Cardiol 11(9):507–515. 10.1038/NRCARDIO.2014.83 24958077 10.1038/nrcardio.2014.83

[13] Obokata M, Olson TP, Reddy YNV, Melenovsky V, Kane GC, Borlaug BA (2018) Haemodynamics, dyspnoea, and pulmonary reserve in heart failure with preserved ejection fraction. Eur Heart J 39(30):2810–2821. 10.1093/EURHEARTJ/EHY268 29788047 10.1093/eurheartj/ehy268 PMC6658816

[14] Reddy YNV, Olson TP, Obokata M, Melenovsky V, Borlaug BA (2018) Hemodynamic correlates and diagnostic role of cardiopulmonary exercise testing in heart failure with preserved ejection fraction. JACC heart fail 6(8):665–675. 10.1016/J.JCHF.2018.03.003 29803552 10.1016/j.jchf.2018.03.003 PMC6076329

[15] Adamson PB et al (2014) Wireless pulmonary artery pressure monitoring guides management to reduce decompensation in heart failure with preserved ejection fraction. Circ Heart Fail 7(6):935–944. 10.1161/CIRCHEARTFAILURE.113.001229 25286913 10.1161/CIRCHEARTFAILURE.113.001229

[16] Dorfs S et al (2014) Pulmonary capillary wedge pressure during exercise and long-term mortality in patients with suspected heart failure with preserved ejection fraction. Eur Heart J 35(44):3103–3112. 10.1093/EURHEARTJ/EHU315 25161181 10.1093/eurheartj/ehu315

[17] Bergström A, Andersson B, Edner M, Nylander E, Persson H, Dahlström U (2004) Effect of carvedilol on diastolic function in patients with diastolic heart failure and preserved systolic function. Results of the Swedish Doppler-echocardiographic study (SWEDIC). Eur J Heart Fail 6(4):453–461. 10.1016/J.EJHEART.2004.02.003 15182771 10.1016/j.ejheart.2004.02.003

[18] Arnold SV et al (2023) Beta-blocker use and heart failure outcomes in mildly reduced and preserved ejection fraction. JACC Heart Fail 11(8 Pt 1):893–900. 10.1016/J.JCHF.2023.03.017 37140513 10.1016/j.jchf.2023.03.017

[19] Lund LH, Benson L, Dahlström U, Edner M, Friberg L (2014) Association between use of β-blockers and outcomes in patients with heart failure and preserved ejection fraction. JAMA 312(19):2008–2018. 10.1001/JAMA.2014.15241 25399276 10.1001/jama.2014.15241

[20] Silverman DN et al (2019) Association of β-blocker use with heart failure hospitalizations and cardiovascular disease mortality among patients with heart failure with a preserved ejection fraction: a secondary analysis of the TOPCAT trial. JAMA Netw Open 2(12):e1916598. 10.1001/JAMANETWORKOPEN.2019.16598 31800067 10.1001/jamanetworkopen.2019.16598 PMC6902757

[21] Flather MD et al (2005) Randomized trial to determine the effect of nebivolol on mortality and cardiovascular hospital admission in elderly patients with heart failure (SENIORS). Eur Heart J 26(3):215–225. 10.1093/EURHEARTJ/EHI115 15642700 10.1093/eurheartj/ehi115

[22] Ambrosio G et al (2011) β-blockade with nebivolol for prevention of acute ischaemic events in elderly patients with heart failure. Heart 97(3):209–214. 10.1136/HRT.2010.207365 21138861 10.1136/hrt.2010.207365

[23] Zheng SL et al (2018) Drug treatment effects on outcomes in heart failure with preserved ejection fraction: a systematic review and meta-analysis. Heart 104(5):407–415. 10.1136/HEARTJNL-2017-311652 28780577 10.1136/heartjnl-2017-311652 PMC5861385

[24] Yamamoto K (2015) β-Blocker therapy in heart failure with preserved ejection fraction: importance of dose and duration. J Cardiol 66(3):189–194. 10.1016/J.JJCC.2015.02.004 25881728 10.1016/j.jjcc.2015.02.004

[25] Yamamoto K, Origasa H, Hori M (2013) Effects of carvedilol on heart failure with preserved ejection fraction: the Japanese diastolic heart failure study (J-DHF). Eur J Heart Fail 15(1):110–118. 10.1093/EURJHF/HFS141 22983988 10.1093/eurjhf/hfs141

[26] Lam PH et al (2018) Role of high-dose beta-blockers in patients with heart failure with preserved ejection fraction and elevated heart rate. Am J Med 131(12):1473–1481. 10.1016/J.AMJMED.2018.07.008 30076815 10.1016/j.amjmed.2018.07.008 PMC10463568

[27] Shah R, Wang Y, Foody JAM (2008) Effect of statins, angiotensin-converting enzyme inhibitors, and beta blockers on survival in patients ≥ 65 years of age with heart failure and preserved left ventricular systolic function. Am J Cardiol 101(2):217–222. 10.1016/j.amjcard.2007.08.050 18178410 10.1016/j.amjcard.2007.08.050

[28] Yndigegn T et al (2024) Beta-blockers after myocardial infarction and preserved ejection fraction. N Engl J Med 390(15):1372–1381. 10.1056/NEJMoa2401479 38587241 10.1056/NEJMoa2401479

[29] Ibrahim J et al (2024) Beta blockers are associated with lower all-cause mortality among HFpEF patients. Clin Res Cardiol 113(6):951–958. 10.1007/s00392-024-02451-0 38695899 10.1007/s00392-024-02451-0

[30] Mebazaa A et al (2022) Safety, tolerability and efficacy of up-titration of guideline-directed medical therapies for acute heart failure (STRONG-HF): a multinational, open-label, randomised, trial. The Lancet 400(10367):1938–1952. 10.1016/S0140-6736(22)02076-1 10.1016/S0140-6736(22)02076-1 36356631

[31] Remme W (2001) Guidelines for the diagnosis and treatment of chronic heart failure. Eur Heart J 22(17):1527–1560. 10.1053/euhj.2001.2783 11492984 10.1053/euhj.2001.2783

[32] Swedberg K et al (2005) Guidelines for the diagnosis and treatment of chronic heart failure: executive summary (update 2005): the task force for the diagnosis and treatment of chronic heart failure of the European Society of Cardiology. Eur Heart J 26(11):1115–1140. 10.1093/EURHEARTJ/EHI204 15901669 10.1093/eurheartj/ehi204

[33] Dickstein K et al (2008) ESC guidelines for the diagnosis and treatment of acute and chronic heart failure 2008: the task force for the diagnosis and treatment of acute and chronic heart failure 2008 of the European Society of Cardiology. Developed in collaboration with the Heart Failure Association of the ESC (HFA) and endorsed by the European Society of Intensive Care Medicine (ESICM). Eur Heart J 29(19):2388–2442. 10.1093/EURHEARTJ/EHN309 18799522 10.1093/eurheartj/ehn309

[34] Dickstein K et al (2010) Focused update of ESC guidelines on device therapy in heart failure: an update of the 2008 ESC guidelines for the diagnosis and treatment of acute and chronic heart failure and the 2007 ESC guidelines for cardiac and resynchronization therapy developed with the special contribution of the Heart Failure Association and the European Heart Rhythm Association. Eur Heart J 31(21):2677–2687. 10.1093/EURHEARTJ/EHQ337 20801924 10.1093/eurheartj/ehq337

[35] Members AF et al (2012) ESC guidelines for the diagnosis and treatment of acute and chronic heart failure 2012: the task force for the diagnosis and treatment of acute and chronic heart failure 2012 of the European Society of Cardiology. Developed in collaboration with the Heart Failure Association (HFA) of the ESC. Eur Heart J 33(14):1787–1847. 10.1093/EURHEARTJ/EHS104 22611136 10.1093/eurheartj/ehs104

[36] Ponikowski P et al (2016) 2016 ESC guidelines for the diagnosis and treatment of acute and chronic heart failure: the task force for the diagnosis and treatment of acute and chronic heart failure of the European Society of Cardiology (ESC). Developed with the special contribution of the Heart Failure Association (HFA) of the ESC. Eur Heart J 37(27):2129–2200m. 10.1093/EURHEARTJ/EHW128 27206819 10.1093/eurheartj/ehw128

[37] Musse M et al (2023) Physician perspectives on the use of beta blockers in heart failure with preserved ejection fraction. Am J Cardiol 193:70–74. 10.1016/j.amjcard.2023.01.050 36878055 10.1016/j.amjcard.2023.01.050 PMC10114214

